# Role of Coenzyme Q10 in Prophylaxis of Myocardial Infarction

**DOI:** 10.7759/cureus.13137

**Published:** 2021-02-04

**Authors:** Iftikhar Ali Shah, Mubeen Memon, Sheeba Ansari, Ratan Kumar, Sultan A Chandio, Shahid H Mirani, Amber Rizwan

**Affiliations:** 1 Internal Medicine, Ghulam Muhammad Mahar Medical College and Hospital, Sukkur, PAK; 2 Pulmonology, Liaquat University of Medical and Health Sciences, Jamshoro, PAK; 3 Internal Medicine, Liaquat University of Medical and Health Sciences, Jamshoro, PAK; 4 Internal Medicine, Civil Hospital Khairpur, Khairpur, PAK; 5 Medicine, Shaheed Mohtarma Benazir Bhutto Medical University Larkana, Larkana, PAK; 6 Surgery, Ghulam Muhammad Mahar Medical College, Sukkur, PAK; 7 Family Medicine, Jinnah Post Graduate Medical Center, Karachi, PAK

**Keywords:** coenzyme q10, prophylaxis, myocardial infarction

## Abstract

Introduction: Coenzyme Q10 (CoQ10) has a potential role in reducing the risk of myocardial infarction by slowing the progression of atherosclerosis and improving ischemia. In this study, we will assess the role of coenzyme Q10 in prophylaxis for reducing myocardial infarction and mortality related to myocardial infarction.

Methods: This open-label two open placebo-controlled randomized clinical trial was conducted in a tertiary care hospital in Sukkur, Pakistan from April 2016 to September 2019. Eight hundred nighty-two (892) patients with clinically diagnosed and documented evidence of hypertension were enrolled in the study from the outpatient department. Participants were randomized into two groups by 1:1 ratio using an online randomizer software, Research Randomizer (https://www.randomizer.org/). Group A received 100 mg coenzyme Q10 daily (coenzyme Q10 group) in addition to standard therapy and group B received standard therapy only (placebo group).

Results: Participants who received coenzyme Q10 had fewer incidence of non-fatal myocardial infarction over 12 months (5.4% vs 8.4%) with relative risk reduction of 2.92 (confidence interval 95%, 0.55-2.76). The number needed to treat to prevent one non-fatal myocardial infarction was 34. Participants who received coenzyme Q10 had fewer incidence of fatal myocardial infarction over 12 months (1.5% vs 3.1%) with relative risk reduction of 1.65 (confidence interval 95%, 0.39-3.69). Number needed to treat to prevent one fatal myocardial infarction was 60.

Conclusion: According to this study, coenzyme Q10 reduced the incidence of fatal and non-fatal myocardial infarctions. Clinicians should consider adding coenzyme Q10 to the treatment regimen of high-risk patients of myocardial infarction. We suggest coenzyme Q10 may be an effective prophylactic agent in patients at risk of myocardial infarction and it may help in reducing burden on the health care system.

## Introduction

Coenzyme Q10 (CoQ10), which is synthesized in the inner mitochondrial membrane, is an essential compound of the human body [1]. CoQ10 is a key component of the electron transport chain in mitochondria necessary for adenosine triphosphate (ATP) production [2]. It also transfers electrons from complexes I and II to complex III in the inner mitochondrial membrane [2].

CoQ10 may have a plausible role in prevention and treatment of heart ailment, as it improves cardiac cellular bioenergetics [3,4]. CoQ10 inhibits progression of atherosclerosis by limiting oxidation of low-density lipoprotein (LDL). It also plays a role in improving ischemia and reduces the risk of reperfusion injury of coronary revascularization [5]. The prevalence and healthcare cost of cardiovascular diseases are increasing every year; hence, it is important that molecules that play a role in preventing cardiovascular diseases should be studied [6]. In this study, we will assess the role of coenzyme Q10 in prophylaxis for reducing myocardial infarction and mortality related to myocardial infarction.

## Materials and methods

This open-label two open placebo-controlled randomized clinical trial was conducted in a tertiary care hospital in Sukkur Pakistan from April 2016 to September 2019. Eight hundred nighty-two (892) patients with clinically diagnosed and documented evidence of hypertension were enrolled in the study from the outpatient department. Ethical review board approval from Ghulam Muhammad Mahar Medical College was taken before enrollment of patients (approval GMMMC/IRB-OfC/2016-82). Patients were enrolled using a consecutive convenient non-probability sampling technique. To nullify the effect of different treatment used in management of hypertension, patients only on angiotensin converting enzyme inhibitor (ACEi) in combination with hydrochlorothiazide for hypertension, rosuvastatin for hypercholesterolemia and aspirin for inhibiting platelet aggregation were enrolled. Participants were randomized into two groups by 1:1 ratio using an online randomizer, Research Randomizer (https://www.randomizer.org). Group A received 100 mg coenzyme Q10 daily twice a day (coenzyme Q10 group) in addition to standard therapy and group B received standard therapy only (placebo group).

The patients' characteristics such as age, gender, history of smoking, blood pressure, previous history for myocardial infarction, and family history for myocardial infarction were noted in the self-structured questionnaire. Patients were followed up for 12 months or for development of myocardial infarction in the outpatient department. Myocardial infarction (MI) was diagnosed based on clinical symptoms, cardiac markers such as troponin, and electrocardiogram changes. Thirty-eight (38) and twenty-nine (29) participants in co-enzyme Q10 and placebo, respectively, were lost to follow up and were not included in the final analysis.

Statistical analysis was done using Statistical Package for Social Sciences (SPSS) version 23 (IBM Corp., Armonk, NY, USA). Continuous variables were analyzed via descriptive statistics and were presented as mean and standard deviation (SD) while categorical variables were presented as percentages and frequencies. Relative risk reduction (RRR) and number needed to treat were calculated via an online calculator (MedCalc Statistical Software; MedCalc Software bv, Ostend, Belgium; https://www.medcalc.org) using 95% confidence interval. Survival probability will be plotted on Kaplan Meier graph. The log rank test was used to calculate the chi-square (X2) for each event time for each group. A p-value of less than 0.05 meant that there is a difference between the two groups and the null hypothesis is void.

## Results

Eight hundred twenty-five (825) participants completed the study, 408 in the coenzyme Q10 group and 417 in the placebo group. There was no difference in demographics and risk factor profile between the two groups (Table 1).

**Table 1:** Characteristics of Participants Abbreviation: SD, standard deviation. NS, non-significant, kg: kilogram, m2: square meter

Characteristics	Coenzyme Q10 Group (408)	Placebo Group (417)	P-value
Age in years (Mean ±SD)	51 ± 14	52 ± 16	NS
Male (%)	234 (57.4%)	248 (59.5%)	NS
Smoking (%)	131 (32.1%)	121 (29.0%)	NS
Diabetes (%)	102 (25.0%)	98 (23.5%)	NS
Hypercholesterolemia (%)	252 (61.8%)	269 (64.5%)	NS
Body Mass Index greater than 25 kg/m^2 ^(%)	201 (49.3%)	199 (47.7%)	NS
Previous history of Acute myocardial Infarction (%)	22 (5.4%)	25 (6.0%)	NS
Family history of Acute Myocardial Infarction (%)	10 (2.5%)	09 (2.2%)	NS

Participants who received coenzyme Q10 had fewer incidence of non-fatal myocardial infarction over 12 months (5.4% vs 8.4%) with relative risk reduction of 2.92 (confidence interval 95%, 0.55-2.76). The number need to treat to prevent one non-fatal myocardial infarction was 34. Participants who received coenzyme Q10 had fewer incidence of fatal myocardial infarction over 12 months (1.5% vs 3.1%) with relative risk reduction of 1.65 (confidence interval 95%, 0.39-3.69). The number need to treat to prevent one fatal myocardial infarction was 60.

**Table 2:** Adverse Outcomes in Both Groups CI: Confidence Interval

Adverse Outcome	Coenzyme Q10 Group (408)	Placebo Group (417)	Relative Risk Reduction (CI, 95%)	Number Need to treated
Fatal Myocardial Infarction	06 (1.5%)	13 (3.1%)	1.65 (0.39-3.69)	60
Non-Fatal Myocardial Infarction	22 (5.4%)	35 (8.4%)	2.92 (0.55-2.76)	34

**Figure 1:**
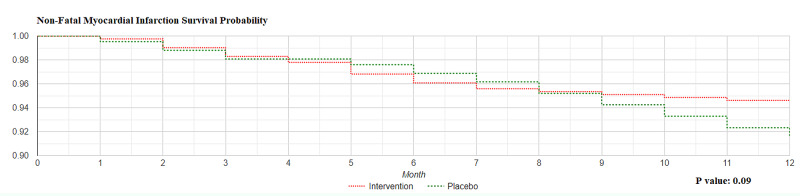
Non-Fatal Myocardial Infarction Survival Probability Intervention group; Co-enzyme Q10 group

As per Kaplan-Meier curve for non-fatal myocardial infarction, the log rank rest for the data in our study was p value = 0.09; thus the two curves were not statistically significantly different (Figure 1).

**Figure 2:**
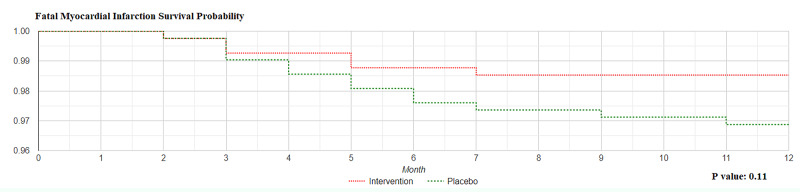
Fatal Myocardial Infarction Survival Probability Intervention group; Co-enzyme Q10 group

As per Kaplan-Meier curve for fatal myocardial infarction, the log rank rest for the data in our study was p value = 0.11; thus the two curves were not statistically significantly different (Figure 2).

## Discussion

According to this study, coenzyme Q10 reduces the risk of fatal and non-fatal myocardial infarction. Eleawa et al. also postulated that CoQ10 may be an effective prophylactic to prevent myocardial infarction (MI) and MI-induced cardiac changes. They found that coenzyme Q10 protects against the reinfarction in myocardial infarction. It also reduces infarct area, inflammation, and oxidative stress while normalizing left ventricular hemodynamics after MI [7]. CoQ10 (ubiquinone) plays an important role to prevent the depletion of ATP as an electron carrier in the mitochondrial respiratory chain and in oxidative phosphorylation [8]. With age, endogenous synthesis of CoQ10 in the body declines, therefore it is recommended that elderly people take CoQ10 as a supplement [9]. Kalen et al. found that 75% of ischemic heart disease patients have low levels of CoQ10 in the plasma [9]. These cardioprotective effects of CoQ10 are due to its antioxidant effect and its ability to generate ATP [10].

Coenzyme Q10 also plays a role in various other cardiovascular disease and events. Coenzyme Q10 reduces admission in heart failure and reduces the episodes of pulmonary edema in heart failure [11]. Munkholm et al. found that coenzyme Q10 improves the stroke index and capillary wedge pressure [12]. Berman et al. found that coenzyme Q10 improves 6 minute walk time in patients with heart failure [13]. Pourmoghaddas et al. in their study found that supplement of coenzyme Q10 is associated with improved ejection fraction [14]. Mortensen et al. found that supplement of coenzyme Q10 reduces the risk of all cause-death, worsening of heart failure, and cardiovascular death [15].

To the best of our knowledge, this is the first study in a local setting that studies the role of prophylaxis against myocardial infarction. The study has its limitation as well. First, since it was single-center study, sample size diversity was reduced. Second, participants were not followed post-MI, hence the role of coenzyme Q10 in preventing MI induced could not be studied in detail.

## Conclusions

In this study, coenzyme Q10 reduced the incidence of fatal and non-fatal myocardial infarctions. Clinicians should consider adding coenzyme Q10 to the treatment regimen of high-risk patients of myocardial infarction. We suggest coenzyme Q10 may be an effective prophylactic agent in patients at risk of myocardial infarction and it may help in reducing the burden on health care systems. Using coenzyme Q10 as a supplement will improve the quality of life of such patients.
